# 

**Published:** 1871-04

**Authors:** 


APRIL, 1871.

### THE AMERICAN JOURNAL OF DENTAL SCIENCE,

EDITED BY

F. J. S. GORGAS, M. D., D. D. S.

PUBLISHED MONTHLY.

$2.50 PER ANNUM.

VOL. 4.—THIRD SERIES.—NO. 12.

BALTIMORE:
SNOWDEN & COWMAN.
LONDON:
TRUBNER & CO., 60 PATERNOSTER ROW.
1870.

PAYABLE IN ADVANCE.

